# Intra-apheresis Cycling to Improve the Clinical Efficacy of Peripheral Blood Stem Cell Donations

**DOI:** 10.1007/s40279-025-02183-9

**Published:** 2025-04-15

**Authors:** Alex J. Wadley, Fendi Pradana, Tarondeep Nijjar, Mark T. Drayson, Samuel J. E. Lucas, Francesca A. M. Kinsella, Phoebe A. Cox

**Affiliations:** 1 School of Sport, Exercise, and Rehabilitation Sciences, University of Birmingham, Birmingham, B15 2TT UK; 2 Institute of Immunology and Immunotherapy, University of Birmingham, Birmingham, UK; 3 Birmingham Centre of Cellular Therapy and Transplantation, Queen Elizabeth Hospital, Birmingham, UK; 4 Nutrition Study Program, Tadulako University, Palu, Indonesia; 5 Clinical Immunology Service, University of Birmingham, Birmingham, UK

## Abstract

Peripheral blood stem cell (PBSC) donation is the primary procedure used to collect haemopoietic stem cells (HSCs) for transplantation in individuals with haematological malignancies. More than 90,000 HSC transplants take place globally each year, and there is an increasing need to guarantee HSC mobilisation, improve tolerability to apheresis, and optimise immune reconstitution. Currently, mobilisation of HSCs depends upon pharmacological agents, with donors inactive during their subsequent apheresis. A successful yield of HSCs is not always achieved, and greater efficiency of collection procedures would improve the donors’ safety and experience, along with the overall functioning of apheresis departments. The mobilisation of immune cells during bouts of exercise has been increasingly studied over the past 40 years. Exercise enriches peripheral blood with HSCs and immune cells such as cytolytic natural killer cells, and these may impact upon collection efficiency and patient outcomes following transplantation. Using exercise in conjunction with routine pharmaceutical agents may meet these needs. This article describes the impact of exercise on the quantity and engraftment potential of HSCs. Given that PBSC collections take on average 3–4 h per day per donor, and often consecutive days to complete, particular attention is paid to adopting interval exercise in this setting. Moreover, practical and safety considerations for allogeneic and autologous donors are discussed. ‘Intra-apheresis cycling’ is proposed as a feasible adjunctive strategy to evoke clinically significant improvements in the quality of the immune graft. Further research is needed to validate this concept in conjunction with routine mobilisation agents.

**Supplementary Information:**

The online version contains supplementary material available at 10.1007/s40279-025-02183-9.

## Key Points

**Table:** 

There is a clinical need to improve the collection of stem cells for transplant for people with blood cancers.
The process of collecting stem cells from a person with blood cancer or a healthy matched individual takes approximately 3–4 h and, often, repeated hospital visits to collect enough cells.
Short periods of cycling interspersed throughout a stem cell collection session could sustain clinically relevant numbers of stem cells and other white blood cells that offset the risk of post-transplant complications.

## Introduction

### Haematopoietic Stem Cell Transplantation

Haemopoietic stem cells (HSCs) are a self-renewing population that reside in the bone marrow of adult organisms. The differentiation of HSCs results in the replenishment of approximately 1 billion mature blood cells in the circulation per day [1]. The regenerative capacity of transplanted HSCs underpins the therapeutic potential of allogeneic stem cell transplantation (allo-SCT) for patients with myeloid and lymphoid haematological malignancies, bone marrow failure syndromes, and inherited haemoglobinopathies, along with autologous stem cell transplantation (auto-SCT) for patients with myeloma and autoimmune disorders [2]. In 2016, 82,718 stem cell transplantations took place worldwide and rates are increasing by approximately 7% each year [3]. Peripheral blood stem cell (PBSC) donation is the most common procedure used to collect HSCs, and this is undertaken by patients requiring auto-SCT or by healthy donors for patients requiring allo-SCT. Issues relating to PBSC collection efficiency and the optimisation of graft composition challenge the deployment of auto-SCT and allo-SCT such that the search for adjunctive strategies to improve the process is of paramount importance.

### Current HSC Mobilisation Strategies

The frequency of HSCs in peripheral blood is very low (0.01–0.1%) compared to bone marrow (1–3%) [4]. Therefore, prior to PBSC collections, 4–5 consecutive days of granulocyte colony-stimulating factor (G-CSF) subcutaneous injections are administered to increase the proliferation of HSCs in the bone marrow and movement into peripheral blood. Plerixafor is the only licensed pharmaceutical agent administered if G-CSF fails to mobilise sufficient HSCs into the circulation [5], thus delaying apheresis. During PBSC donation, the HSC-rich leukocyte fraction is isolated by processing the donor’s blood volume two to six times over 3–4 h, and this can be associated with significant side effects. Extracorporeal processing of large blood volumes (10–30 L [6]) requires the use of anticoagulant agents, commonly citrate, which can lead to hypocalcaemia [7], whilst blood volume losses of up to 15% can cause hypotension and anaemia [8]. Furthermore, administration of G-CSF can elicit bone pain, headaches, fatigue, and, rarely, splenic rupture or thromboembolism [9].

### Allogeneic Donations

The clinical efficacy of apheresis is solely defined by the degree of HSC mobilisation, which is largely successful for allogenic donations (< 5% failure [10]). Achieving an HSC dose of > 4 × 10^6^/kg reduces the risk of engraftment failure in recipients with myeloid and lymphoid leukaemias [11]. However, a major health complication following allo-SCT is graft versus host disease (GvHD), in which epithelial barrier tissues (e.g. skin, gastrointestinal tract, and liver) are damaged. The incidence of acute GvHD ranges between 20 and 50% depending upon the style of transplant deployed, whilst chronic GvHD ranges from 30 to 70% [12]. Even in allo-SCT undertaken with human leukocyte antigen (HLA)-‘matched’ donors, acute and chronic GvHD results in a transplant-related mortality rate of 15% at 1–2 years [13]. Although not evaluated clinically, the composition of other cells within the donated graft can impact the risk of GvHD and other clinical endpoints following allo-SCT. Higher numbers of monocytes [14], natural killer (NK) cells [15]), gamma delta (γδ) [16], and regulatory T (T_reg_) cells [17] have been associated with a lower risk of GvHD [18]. Conversely, higher numbers of memory T cells have been associated with greater risk [19], as histocompatibility differences between graft and host T cells may result in alloreactive damage.

### Autologous Donations

Mobilisation failure is far more common with autologous donations. After myeloablative, or multiple lines of targeted cancer treatments, failure rates of up to 40% have been reported by the American Society for Blood and Marrow Transplantation, resulting in lengthy donation periods (i.e. repeated hospital visits) and/or sub-optimal HSC doses [20]. Infusion of < 1.5–2.5 × 10^6^ HSCs/kg leads to poor reconstitution of haemopoiesis and increased risk of graft failure [21] due to delayed neutrophil (< 0.5 × 10^9^/L) and platelet recovery (< 20 × 10^9^/L). The composition of other cell types in the graft, e.g. T_reg_ cells [22] and NK cells [23] may also impact upon clinical outcomes. The commonest indication for auto-SCT is multiple myeloma, where it is the current gold standard treatment for patients who respond to first-line treatment [24]. Novel triplet and quadruplet therapies have greatly improved outcomes for patients with multiple myeloma [25], but adversely impact PBSC collection efficiency following G-CSF [26], highlighting an unmet need to improve procedures [27].

### Peripheral Blood Stem Cell Donation: A Sedentary Process

Issues with both autologous and allogeneic PBSC donations result in delays in treatment, poor engraftment of HSCs, increased symptom burden and risk, and cost/resource implications for healthcare systems. There is a clinical need to implement new approaches to improve HSC mobilisation, immune reconstitution, and tolerability to the PBSC donation process. Donors sit relatively immobile for 3–4 h, frequently over consecutive days to obtain the desired HSC dose. During the first era of stem cell transplantation almost 50 years ago, it was reported that just 4 min of vigorous stair climbing increased the concentration of HSCs in peripheral blood [28]. The authors concluded that ‘the procedures described could be exploited in the use of leukapheresed nucleated cells as a source of stem cells for bone marrow transplantation’. A body of work has since verified these findings and established that exercise evokes parallel increases in differentiated immune cell subsets with potent effector functions [29, 30]. These changes to immune composition could therefore enhance graft quality and quantity during PBSC collections. Bouts of exercise have been previously used in clinical settings to improve clinical endpoints [31, 32] and achieve health economic benefits [33]. However, feasible protocols for use during PBSC collections have yet to be designed or evaluated, particularly in conjunction with routine pharmacological agents. This is critical given that exercise does not result in a sufficient increment in HSC concentration to meet the threshold required to initiate apheresis (> 10 cells/μL [34]).

## Objectives

This article discusses the existing literature on the topic of exercise-induced potentiation of HSCs in peripheral blood, with a focus on the mechanisms underpinning HSC and immune cell mobilisation with exercise (Sect. 4.1). This facilitates a focused discussion on how intermittent periods of cycling might be implemented over a typical donation period to sustain both HSC and effector immune cell concentrations in peripheral blood (Sects. 4.2 and 4.3). Based on the existing literature, the concept of ‘intra-apheresis cycling’ is introduced (Sect. 4.4) and the potential impact of this on graft quality and clinical endpoints. All studies reviewed have examined the effects of exercise on HSCs in individuals not undergoing G-CSF therapy. To address future research in this area, the feasibility of implementing intra-apheresis cycling for both allogeneic and autologous donors is discussed (Sect. 4.5).

## Methods

### Literature Searches

Given the novelty of the proposed topic and need to integrate literature from different disciplines to support the writing, a narrative review is appropriate at this time. To guide the primary topic of how single bouts of exercise affect peripheral blood concentrations of HSCs, we used the Scale for the Assessment of Narrative Review Articles [35]. Search terms included were ‘Haemopoietic stem’ AND ‘Progenitor cell’ AND ‘Exercise’ AND ‘acute’, generating 54 (PubMed), 39 (Medline), and 40 (Web of Science) search items, of which 23 were finally included. A list of final articles is included in Table S1 (see the electronic supplementary material), with only original research articles included in English language. It is acknowledged that this may have excluded information evolving across the globe.

### Classification of HSCs

A notable point when comparing studies investigating changes in HSCs in response to bouts of exercise is the variation in concentrations reported between studies [36]. Specifically, across the existing literature, we calculated that post-exercise HSC concentrations (cells per µL, 6.19 ± 8.75) were significantly greater than rest concentrations (4.34 ± 6.34), but the coefficients of variance were 146% and 141%, respectively, indicating high inter-study variance (Fig. S1 and Table S1; see the electronic supplementary material). This can be explained by differences in methodological approaches, including (1) analysing whole blood or purified peripheral blood mononuclear cells (PBMCs) for cell surface antibody staining, (2) use of single- or double-platform quantitative methods, and notably, (3) the criteria used to define HSCs. The gold standard method for enumerating HSC concentration in blood has been outlined by the International Society of Hematotherapy and Graft Engineering (ISHAGE) since 1998 [37], but this has seldom been adopted in these studies. The ISHAGE method is a single-platform approach using whole blood that has a within- and between-laboratory variance of < 5% and < 9%, respectively [37], whereas between-laboratory variance for the double-platform method is reported as high as 21% [38]. Under ISHAGE guidance, HSCs are defined as cluster of differentiation (CD)34^+^CD45_dim_SSC_low_ using a strict Boolean gating strategy. This approach also incorporates haemopoietic progenitor cells (HPCs) into the criteria, thus strictly defining these cells as haemopoietic stem and progenitor cells (HSPCs). Furthermore, many studies report CD34^+^ counts only (incorporating further diversity in progenitor cell populations) or define HSCs using different criteria (CD34^+^CD33^+^, CD34^+^CD38^+^, CD34^+^HLA-DR^+^) [39], which may inflate or diminish true HSC counts. In this review, interpretations are made using all cell populations and referred to as HSCs for continuity. This facilitates discussion on the clinical relevance of this work, but the exact criteria used in each study are outlined in Table S1.

### Equality, Diversity, and Inclusion Statement

The author group consists of junior, mid-career, and senior researchers of different genders and ethnicities, residing in the UK and Indonesia. Author disciplines included immunology, medicine, and sports science. The article evaluates how adapting an established clinical procedure might improve the collection of HSCs from healthy people and those with a range of illnesses. The influence of age and biological sex is considered in Sect. 4.5; however, we acknowledge we did not discuss the effects of race/ethnicity or socioeconomic status.

## Discussion

### Peripheral Blood HSC Concentrations After Exercise

#### Biphasic Change

**Fig. 1:**
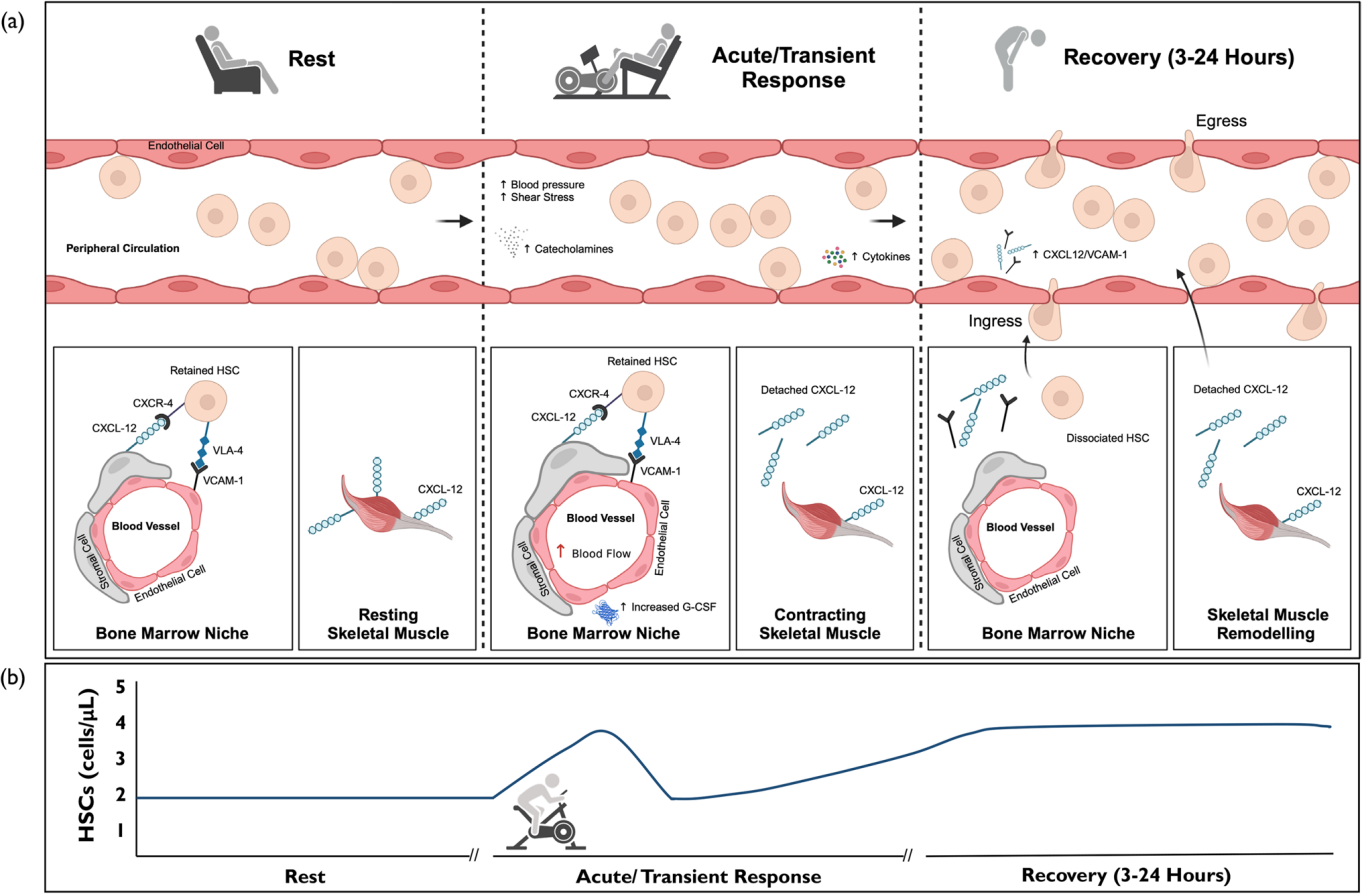
Time course and mechanisms underpinning the biphasic increase in peripheral blood HSC concentrations. **a** Illustrates the interactions between peripheral blood (depicted as 1 µL volume), the bone marrow niche, and skeletal muscle at rest (left), during the transient increase in response to exercise (middle), and during recovery (right). HSC concentration increases within the peripheral blood compartment during exercise as cells are mobilised from marginal pools via physiological (blood pressure and shear stress) and biochemical mechanisms (catecholamines and cytokines). A secondary increase follows between 3 and 24 h later as HSCs egress from the bone marrow into the blood compartment, replacing HSCs (and other immune cells) that egress from peripheral blood to sites of inflammation. This increase may result from (1) increased blood flow to the bone marrow, (2) increased G-CSF production within the bone marrow niche, (3) reduced CXCR-4- and VLA-4-dependent HSC adhesion to the bone marrow stroma, and (4) chemoattractant gradients promoting egress of HSCs into peripheral blood. There are data to indicate that the latter is governed by the release of chemokines from skeletal muscle that may draw HSCs into peripheral blood, although other tissues could contribute. **b** Illustrates the predicted time course of HSC concentrations in peripheral blood in response to bouts of exercise based on the available studies. This will vary depending on the intensity and duration of the bout and degree of muscle damage. *CXCL-12* C-X-C motif chemokine 12, *CXCR-4* C-X-C chemokine receptor type 4, *G-CSF* granulocyte-colony stimulating factor, *HSC* haemopoietic stem cell, *VCAM-1* vascular cell adhesion molecule 1, *VLA-4* very late antigen-4

Cycling, running, stair climbing, and rowing exercise evoke two- to threefold increases in peripheral blood HSC concentrations after completion [40, 41]. This is first characterised by a transient initial increase (≈ 5 min [42]) that is driven more by exercise intensity [43, 44] than by duration [45, 46]. A secondary increase ≈ 3 h after exercise cessation follows this [40, 47], and is of similar magnitude but prolonged by a higher degree of muscle damage [40]. This biphasic change in peripheral blood HSC numbers is underpinned by distinct physiological and biochemical mechanisms that accompany exercise (Fig. 1).

#### Acute/Transient Change

Increases in cardiac output, blood pressure, and blood flow result in haemodynamic shear stress that rapidly demarginates all leukocyte subsets from marginal pools of the circulation and pulmonary, splenic, or skeletal muscular reservoirs in a non-specific manner [48]. Exercise-induced leukocytosis is not uniform across all subsets, with cells exhibiting greater effector function, antigen experience, and/or differentiation preferentially mobilised due to their higher cell surface expression of β2-adrenergic receptor (β2-AR) [49]. For example, cytolytic NK cells (CD56_dim_), non-classical monocytes, γδ T cells, and effector CD8^+^ T cells have higher β2-AR expression than other immune cell subsets such as HSCs [49, 50], resulting in greater fold changes during exercise [49, 51]. Notably, CD56_dim_ NK cell concentrations increase up to tenfold during high-intensity exercise [30], whereas only two- to threefold increases in HSCs are observed at the same intensity. The increase in HSC numbers during exercise therefore mirrors the total leukocyte pattern [52]. Nevertheless, direct evidence in humans indicates that HSC mobilisation is driven, in part, by the effects of β2-AR signalling [43], with interleukin-6 [53], G-CSF [40], and growth factors [54] possibly providing chemoattractant cues for HSC demargination into blood. These mechanisms underpin the intensity dependent relationship between exercise and HSC mobilisation. This is best illustrated by a study reporting a ≈twofold increase in HSC concentration after 15 min of running at 15% above the lactate threshold (LT), and no change after 90 min at 5% below the LT [43]. More recent work reported greater HSC mobilisation after just 2 × 2 min bouts of interval cycling versus 30 min of moderate-intensity continuous cycling [55].

#### Secondary Influx from Bone Marrow in Recovery

Following β2-AR-dependent mobilisation of cells with a tissue homing phenotype, these cells are then redistributed from peripheral blood under the actions of glucocorticoids [55] during the exercise recovery period. Differentiated lymphocytes and NK cells migrate rapidly to lymphoid tissues [56], whereas monocytes migrate to skeletal muscle tissue to facilitate tissue remodelling [48]. Egress of naïve T cells from the thymus [57] and immature monocytes and neutrophils from the bone marrow are believed to then rejuvenate these cell populations in peripheral blood. Data in humans [40, 47] and animals [58] also indicate that HSCs egress from the bone marrow into peripheral blood to accompany these dynamic whole-body changes.

After birth, HSCs are produced in bone marrow and retained via interactions between anchored secreted proteins on bone marrow stromal cells, notably, very late antigen-4 (VLA-4) and C-X-C chemokine receptor type 4 (CXCR-4). Dissociation of these interactions results in an influx of HSCs into peripheral blood, with the latter targeted by plerixafor during PBSC donations. Interestingly, there is evidence from animal studies to indicate that exercise may disrupt these receptor/ligand interactions within the bone marrow niche directly through local G-CSF production [58]. HSCs may also be attracted into the circulation via altered chemokine gradients [40, 43, 59, 60] (Fig. 1). These factors, as well as an increase in blood flow to the bone marrow during exercise [61, 62], are possible mechanisms explaining the secondary increase in peripheral blood HSCs observed ≥ 3 h after aerobic exercise [40, 47] and for at least 24 h after muscle damaging exercise [40]. HSCs may replace circulating apoptotic blood cells or migrate to sites of tissue damage and inflammation, such as skeletal muscle and the spleen, to facilitate repair processes [63].

The available evidence indicates that exercise elicits a biphasic increase in HSC concentrations, with exercise intensity being a key mediator of the initial response, and muscle damage the latter. Parallel increases in effector immune cells collectively cause marked changes to the composition of the immune cell fraction within peripheral blood.

### Effect of Interval Cycling on Peripheral Blood HSC Concentrations

#### Concept Versus Reality

PBSC collections take between 3 and 4 h, and demargination of HSCs and other immune cells during high-intensity exercise is a transient response (≈ 5–20 min depending on the cell type). Sustained exercise at high intensity, particularly at or near to maximal exertion, may be feasible in ‘healthy’ allogenic donors that undertake exercise regularly, but less plausible for those in the general population that do not, and certainly unfeasible for autologous donors burdened with cancer-related morbidity and therapy. Intermittent periods of exercise (or ‘intervals’) at an achievable intensity may be the only feasible approach to maximise concentrations of HSCs and other clinically relevant cells over a 3- to 4-h period.

#### Interval Exercise and HSCs

Interval exercise involves working briefly at moderate-to-high intensity, interspaced with rest intervals and an overall lower exercise volume compared to sustained, continuous exercise. These intervals can vary, from 30 s at maximal intensity [64] to 4 min at a high but more manageable intensity [65]. The latter have been used in clinical settings, notably for cardiac rehabilitation [65], and are considered more appropriate for much of the population [66]. The available data indicate that sub-maximal interval exercise < 30 min (e.g. 2- to 4-min intervals interspersed with rest periods and totalling as little as 4 min of work [67]) can potentiate peripheral blood HSC concentrations in a biphasic manner [47, 60], although one study did report no change after 10 × 1-min cycling bouts at 90% of maximum heart rate [52]. Future studies are needed to empirically determine the optimal balance between exercise intensity and duration. Furthermore, it is important to establish how feasible and clinically effective these protocols are within a typical PBSC session (e.g. 3–4 h).

#### Clinical Thresholds

The typical threshold for collection of HSCs during PBSC donations is a blood concentration of > 10 cells/µL (ideally > 20). This is with the aim of achieving a collection of 2–5 million HSCs/kg of the recipient’s body weight by apheresis for successful engraftment and rapid recovery of neutrophil and platelet counts [68]. Exercise of high intensity in the absence of G-CSF elicits peak concentrations of ≈ 5 cells/μL in peripheral blood [40, 41, 43, 44, 52, 54, 69], which equates to an estimated HSC dose of ≈ 320,000 HSCs/kg^1^. This is inferior to the 10–150 cells/μL that can be achieved following 5 days of G-CSF therapy, which is also sustained over a number of days [70]. It is therefore important to consider whether interval exercise could offer any meaningful clinical value.

### Scope for Interval Exercise to Impact Graft Quality?

#### HSCs

G-CSF therapy promotes HSC proliferation in the bone marrow, resulting in high numbers entering peripheral blood available for collection by apheresis. Donor blood volume is processed three to six times during apheresis to help dislodge HSCs adhering to the endothelium in marginal pools of the circulation [71]. Therefore, the physiological stress of exercise could demarginate HSCs for collection in the graft. It is noteworthy that infusion of low-dose beta-agonists does not elicit leukocytosis of the same magnitude as exercise [30]. These data advocate the use of exercise over further pharmacological intervention, due to the combined actions of β2-AR signalling, haemodynamic shear forces, and other chemoattractant cues. Variation in apheresis procedures are known to impact HSC collection efficiency; notably, larger processed blood volumes and more cycles of apheresis [72]. With cardiac output increasing from ≈ 4–5 L/min at rest to ~ 20 L/min during maximal exercise, repeated surges in blood flow would likely facilitate rather than compromise collection efficiency during apheresis.

#### Immune Graft Composition

In addition to HSC enrichment with interval exercise, higher numbers of effector and regulatory immune cells in the graft could impact upon several clinical endpoints. For example, three- to fourfold increases in CD56_dim_ NK and cytotoxic T cell concentrations have been reported following 60- to 90-s intervals at 85–90% maximal oxygen consumption [29, 73]. These concentrations are sustained for 20 min following interval exercise [74], but their high tissue homing potential results in numbers falling below resting concentration within 60 min of exercise cessation. Studies have also reported increases in T_reg_ [47] and monocyte [75] concentrations immediately after interval exercise. Importantly, these responses are observed in people with solid [76] and liquid tumours [77] after as little as 10 min of cycling. These data therefore provide relevance to both autologous and allogeneic donations. NK cells are the first donor-derived cells to recover after allo-SCT, and more rapid reconstitution has been associated with better survival outcomes [78]. The three- to fourfold increase in CD56_dim_ NK cells after interval exercise [29] might offer protection against disease relapse and GvHD [12]. It must be noted that higher numbers of memory and effector T cells may increase risk of GvHD following allo-SCT [18], and this warrants careful examination in future studies.

The available evidence indicates that conventional interval exercise protocols (< 30 min) enrich peripheral blood with higher numbers of HSCs and effector immune cells, which are primed to assist with haemopoietic engraftment more than cells isolated when at rest. However, there are no data at present to indicate whether intervals interspersed over ≈ 3–4 h can sustain concentrations of these cells, and whether this is clinically viable.

### Intra-apheresis Cycling

**Fig. 2:**
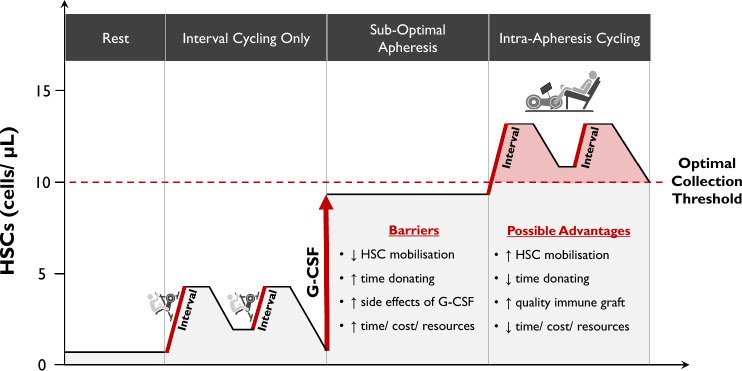
Model of ‘intra-apheresis cycling’ based on existing data and knowledge of how interval exercise elicits changes in immune cell composition. The figure illustrates the concentration of HSCs at rest and during and after interval exercise, in response to G-CSF therapy (without exercise) when apheresis is sub-optimal, and during ‘intra-apheresis cycling’. At rest, HSCs circulate in low concentrations (1–2 cells/μL) in peripheral blood, and cycling increases this ≈ twofold (3–4 cells/μL). G-CSF promotes proliferation of HSCs in the bone marrow and sustained concentrations in peripheral blood, but barriers can result in poor HSC collection rates (< 10 cells/μL). By combining prior G-CSF treatment with intermittent periods of cycling during apheresis, marginated HSCs may be mobilised to expedite PBSC collections, improve graft quality (enrichment with effector immune cells), and improve clinical endpoints. These benefits may be equally applicable to donors with HSC concentrations above the collection threshold, thus improving engraftment potential. *G-CSF* granulocyte colony-stimulating factor, *HSC* haemopoietic stem cell, *PBSC* peripheral blood stem cell

By employing intermittent periods of cycling during PBSC donations, we have coined the term ‘intra-apheresis cycling’. This is a simple, but innovative concept that exploits fundamental human physiological responses to enrich peripheral blood with more clinically relevant cells that can be harvested for patients. The benefits of intra-apheresis cycling have potential to be widespread across all aspects of the PBSC procedure, and these are outlined in Fig. 2. They include enhancing graft quality through enrichment of HSCs and effector immune cell numbers, expediting the apheresis session and relieving donor burden. Improvements in engraftment success and clinical endpoints (e.g. survival, incidence of GvHD, and disease relapse) are also permissible, although the enrichment of peripheral blood with potentially alloreactive cell types needs further investigation.

### Feasibility of ‘Intra-apheresis Cycling’?

#### Safety and Logistical Considerations

The ‘optimal’ physiological response to elicit changes to the immune graft needs to be balanced against the safety of the donor, notably patients during ill-health or undergoing immunosuppressive treatments. Furthermore, allogeneic donors with little exercise experience may struggle with aspects of intra-apheresis cycling. Issues for all donors may include performing intervals over a 3- to 4-h period, physiological strain of the cycling, muscle/joint discomfort, and a lack of enjoyment/compliance. Protocols should be designed with substantially longer rest periods than conventional interval exercise, and suit donors’ exercise capacity rather than a ‘one-size fits all’ approach. G-CSF treatment can cause mild side effects, notably bone pain, headaches, and fatigue [79], and therefore patient safety and medicolegal implications regarding the ‘normal’ volume and intensity of exercise are notable considerations. The safety and benefits of undertaking programmes of exercise before and after HSC transplantation have previously been evaluated [80, 81]. Patients with a range of malignancies, including multiple myeloma, have undertaken aerobic or strength-based exercise programmes during and after cancer therapy regimens. Adherence to these programmes has been high (≈ 85%), and significant improvements in quality of life, fatigue status, and health outcomes (e.g. immunological measures and body composition) have been reported in inpatient [82–85] and outpatient settings [86–89]; however, effects on engraftment and transplant-related outcomes are presently not clear [81]. The former is important given the physical and emotional strain that transplants have on patients’ well-being. A recent study specifically evaluated the feasibility, adherence, and safety of undertaking regular high-intensity interval exercise (HIIE: 2 times per week, for 4–12 weeks) to improve the physical capacity of patients with a range of malignancies prior to allo-SCT [90]. Adherence to HIIE (8 × 1-min bouts at 90–100% of peak power output) was very high (92%), resulting in significant improvements in aerobic fitness prior to transplant. Although encouraging, recruitment for this study was reported to be challenging, and therefore demonstrating clinical efficacy and safety of intra-apheresis cycling will be necessary to improve patient uptake and gain support from health care professionals. The highest numbers of HSCTs are performed in high resource regions, but the incidence is growing at a greater rate in resource-constrained regions, such as Africa and the East Mediterranean region. Bone marrow registries continue to be established globally; however, issues regarding donor availability and drug shortage provide obstacles for expanding these programmes. It is therefore likely that the feasibility of intra-apheresis cycling would be lower in certain parts of the world where healthcare support is less resourced [91].

#### Exchange of HSCs Between Peripheral Blood and Tissues

Periods of rest between each interval of intra-apheresis cycling would undoubtedly be longer than conventional interval exercise protocols if performed over a 3- to 4-h period, thus impacting the exchange of cells between the peripheral blood compartment and lymphoid tissues (e.g. gut and lungs). Concentrations of lymphocytes and monocytes have been reported to significantly decrease within as little as 3 min after exercise cessation, with more rapid rates of egress observed for cells with higher effector functions (NK > CD8^+^  > γδ T cells > CD4^+^) [92]. These cells are rapidly remarginalised within the circulation, with some cells then migrating to lymphoid tissues between 1 and 3 h later. During this period, HSCs and immature granulocytes are released from the bone marrow to restore peripheral blood immune cell concentrations [40, 58, 93]. It is important to consider that the concentrations of these cell types would reflect the net balance of ingress and egress from peripheral blood over 3–4 h of intra-apheresis cycling. Sustaining concentrations over this period for potential harvest would likely depend on how frequently intervals were prescribed to promote shear stress and β2-AR-dependent ingress > egress, with the latter influenced by rest duration.

#### Predictive Variables of HSC Mobilisation

A range of individuals across the population undertake PBSC donations, ranging in health status and fitness. Consequently, there has been a breadth of research that has been undertaken to establish variables that predict collection efficiency of HSCs during apheresis. Whereas factors such as biological sex [94], age [95], previous myeloablative treatments [95], and comorbidities (e.g. diabetes) [96] mediate mobilisation failure after G-CSF therapy, the strongest predictor of successful HSC collection is high pre-procedure peripheral blood HSC count [20]. Interestingly, a small number of studies have reported independent positive associations between resting HSC count and physical activity levels [97], peak oxygen uptake ($\dot{V}$O_2peak_) [98], and training status [45]. A recent study by Ojala et al. (2024) reported that exercise training elicits metabolic adaptations within the bone marrow of humans [99], which could conceivably modulate the production and release of HSCs into peripheral blood. There are also data in animal models indicating that exercise training can increase the number of HSCs in the bone marrow niche without compromising function [81]. Whether this translates into improved HSC collection efficacy in humans is unclear, with one study reporting marginally higher (*P* > 0.05) numbers of collected HSCs (cells/kg) in autologous donors who had undertaken a period of exercise training prior to apheresis, compared to control [100]. Further work is needed to determine the relationship between donors’ training background/fitness and HSC dose after apheresis. In the absence of G-CSF therapy, a recent meta-analysis concluded that variation in HSC mobilisation during exercise is multifaceted [39]. With the proposed addition of cycling to the PBSC collection procedure, determining variables that predict HSC mobilisation in this setting will be important to identify donors most likely to benefit from intra-apheresis cycling.

#### Health Economics

The myriad of economic strains that healthcare systems experience, including staff time, demand for apheresis slots, and pharmaceutical agent costs could be offset by purchasing a cycling ergometer with long durability. Similar ergometers have been implemented in clinical settings previously. For example, patients with chronic kidney disease exhibited improved dialysis efficiency [31], improved health outcomes [32], and, in turn, lower healthcare costs [33] by undertaking supine cycling during dialysis. The running costs of ‘intra-apheresis cycling’ must therefore be leveraged against the health benefits by carrying out cost–benefit analyses.

## Conclusion

Based on our knowledge of exercise-induced potentiation of HSCs and other immune cell types, intra-apheresis cycling may offer a safe, feasible, and economically viable adjuvant therapy to sustain the numbers of these cells during PBSC donations. With an unmet clinical need to improve PBSC donations for both autologous and allogenic transplantation, work in this area should be prioritised to evaluate its clinical effectiveness; however, there is much to be done to facilitate this. This centres on addressing how intra-apheresis cycling can safely complement standard pharmacological agents, such as G-CSF, as well as considering the acceptability for donors and the economic impact on healthcare systems.

## Supplementary Information

Below is the link to the electronic supplementary material.

Supplementary file1 (DOCX 80 KB)

## Footnotes

1 The average height and weight of a male in the UK is 1.77 m and 83.9 kg equating to an estimated total blood volume of 5.4 L. The average height and weight of a female in the UK is 1.61 m and 70.2 kg respectively equating to an estimated total blood volume of 4.4 L. Estimated calculations of cells/kg were based on the peripheral blood concentration of HSCs and Nadler’s equation for calculating total blood volume [101]
