# Supplementary material for: Intra-apheresis Cycling to Improve the Clinical Efficacy of Peripheral Blood Stem Cell Donations
Source: Sports Med. 2025 Apr 15;55(5):1085–96. doi: 10.1007/s40279-025-02183-9 (PMC12106142; doi:10.1007/s40279-025-02183-9)
Supplement: Supplementary file 1 — Supplementary file1 (DOCX 80 KB) [file 40279_2025_2183_MOESM1_ESM.docx]

**Supplementary Materials: Wadley (2024)**

**Supplementary Figure 1**

**
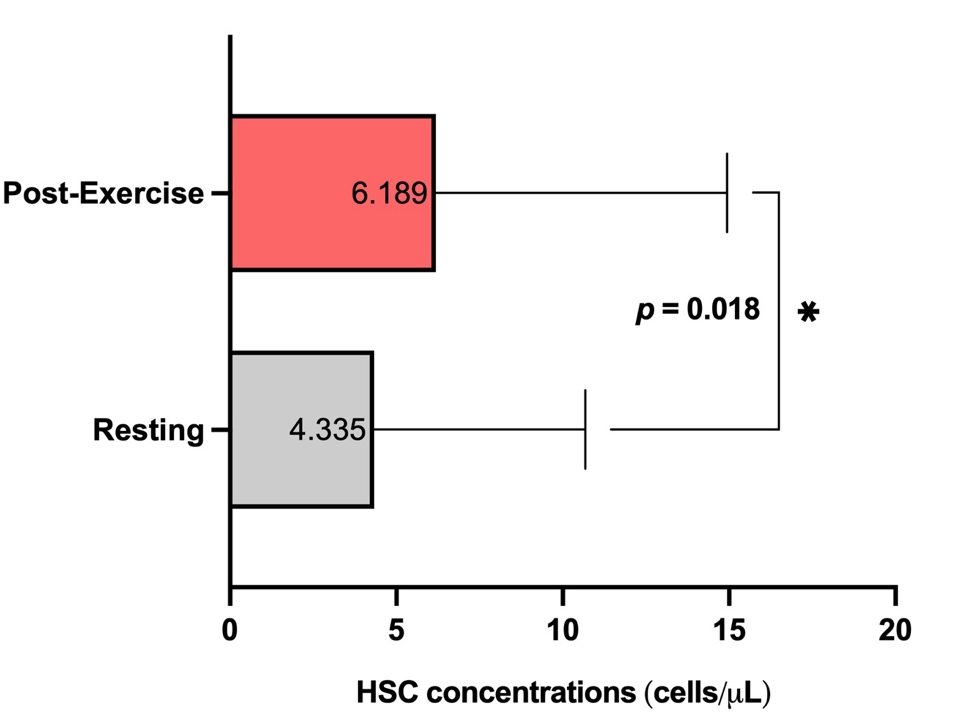
**

**Fig. S1:** Graphical depiction of the exercise-induced HSC response and large degree of variance across 21 independent investigations with available data identified from literature searches of ‘Haemopoietic stem’ AND ‘Progenitor cell’ AND ‘Exercise’ AND ‘acute’ using PubMed, Medline, and Web of Science (see Table S1). HSC concentrations at ‘Rest’ (grey bar) and immediately ‘Post-Exercise’ (red bar) are reported. Values are mean (+ standard deviation). * indicates significant difference between Rest and Post-Exercise: *P = 0.018. Note: HSC concentrations were estimated from figures in 2 studies and data from 2 studies were not available.

**Table S1**

A summary of the articles recovered from the search terms ‘Haemopoietic stem’ AND ‘Progenitor cell’ AND ‘Exercise’ AND ‘acute’ from PubMed (54), Medline (39), and Web of Science (40). A total of 23 original research articles published in English language were the used to guide the primary topic of this article.

| Reference | Title | HSC Classification | Journal |
| --- | --- | --- | --- |
| Pradana, F (2024) | Brief cycling intervals incrementally increase the number of hematopoietic stem and progenitor cells in human peripheral blood | CD34+/CD45dim/SSClow | Front Physiol |
| Niemiro, GM (2017) | Kinetics of circulating progenitor cell mobilization during submaximal exercise | CD34+/CD45low/  CD38- | J Appl Physiol |
| Kröpfl, JM (2021) | Acute exercise-induced glycocalyx shedding does not differ between exercise modalities, but is associated with total antioxidative capacity | CD34+ CD45dim | J Sci Med Sport |
| Kröpfl, JM (2020) | Exercise-Induced Circulating Hematopoietic Stem and Progenitor Cells in Well-Trained Subjects | CD34+ CD45dim | Front Physiol |
| Schmid, M (2020) | Acute Exercise-Induced Oxidative Stress Does Not Affect Immediate or Delayed Precursor Cell Mobilization in Healthy Young Males | CD34+ CD45dim SSClow | Front Physiol |
| Joshi, S (2020) | Blood flow restriction exercise stimulates mobilization of hematopoietic stem/progenitor cells and increases the circulating ACE2 levels in healthy adults | CD34+ CD45dim | J Appl Physiol |
| Kröpfl, JM (2020) | Acute Exercise in Hypobaric Hypoxia Attenuates Endothelial Shedding in Subjects Unacclimatized to High Altitudes | CD34+ CD45dim | Front Physiol |
| Kröpfl, JM (2019) | Acute Exercise-Induced Circulating Haematopoietic Stem and Progenitor Cells in Cardiac Patients - A Case Series | CD34+ CD45+ | Heart Lung Circ |
| Niemiro, GM (2018) | Circulating Progenitor Cell Response to Exercise in Wheelchair Racing Athletes | CD34+ CD45dim | Med Sci Sports Exerc |
| Agha, NH (2018) | Vigorous exercise mobilizes CD34+ hematopoietic stem cells to peripheral blood via the β2-adrenergic receptor | CD34+ | Brain Behav Immun |
| Strömberg, A (2017) | Exercise-induced upregulation of endothelial adhesion molecules in human skeletal muscle and number of circulating cells with remodelling properties | CD34+ CD45dim | J Appl Physiol |
| Krüger, K (2016) | Apoptosis of T-Cell Subsets after Acute High-Intensity Interval Exercise | CD34+ CD45+ | Med Sci Sports Exerc |
| Waclawovsky, G (2016) | Exercise on Progenitor Cells in Healthy Subjects and Patients with Type 1 Diabetes | CD34+ CD45dim | Med Sci Sports Exerc |
| Krüger, K (2015) | Progenitor cell mobilization after exercise is related to systemic levels of G-CSF and muscle damage | CD34+ CD45+ | Scand J Med Sci Sports |
| Riddell, NE (2015) | Progenitor cells are mobilized by acute psychological stress but not beta-adrenergic receptor agonist infusion | CD34+ CD45low SSClow | Brain Behav Immun |
| Thijssen, DH (2006) | Haematopoietic stem cells and endothelial progenitor cells in healthy men: effect of aging and training | CD34+ | Aging Cell |
| Morici, G (2005) | Supramaximal exercise mobilizes hematopoietic progenitors and reticulocytes in athletes | CD34+ | Am J Physiol Regul Integr Comp Physiol |
| Rehman, J (2004) | Exercise acutely increases circulating endothelial progenitor cells and monocyte-/macrophage-derived angiogenic cells | CD133+ VE-Cadherin- | J Am Coll Cardiol |
| Bonsignore, MR (2002) | Circulating hematopoietic progenitor cells in runners | CD34+ | J Appl Physiol |
| Nederveen, JP (2020) | Hematopoietic stem and progenitor cell (HSPC) mobilization responses to different exercise intensities in young and older adults | CD34+ | J Sci Sport Exerc |
| Baker, JM (2017) | Aerobic exercise in humans mobilizes HSCs in an intensity-dependent manner | CD34+ | J Appl Physiol |
| Bonsignore, MR (2010) | Hemopoietic and angiogenetic progenitors in healthy athletes: different responses to endurance and maximal exercise | CD34+ | J Appl Physiol |
| Wardyn, GG (2007) | Effects of exercise on hematological parameters, circulating side population cells, and cytokines | CD34+ | Exp Hematol |
